# Clinical characteristics and severity markers in hospitalized COVID-19 patients from western Mexico: a comparative analysis of Delta and Omicron variants

**DOI:** 10.3389/fpubh.2024.1425372

**Published:** 2024-08-30

**Authors:** Hazael Ramiro Ceja-Gálvez, Cristian Oswaldo Hernández-Ramírez, Alejandra Natali Vega-Magaña, Jorge Hernández-Bello, Kevin Javier Arellano-Arteaga, Francisco Javier Turrubiates-Hernández, Diana Lourdes Padilla-Borquez, José Francisco Muñoz-Valle

**Affiliations:** ^1^Institute of Research in Biomedical Sciences, Centro Universitario de Ciencias de la Salud (CUCS), University of Guadalajara, Guadalajara, Jalisco, Mexico; ^2^Department of Internal Medicine, Hospital Civil de Guadalajara Dr. Juan I. Menchaca, Guadalajara, Jalisco, Mexico

**Keywords:** SARS-CoV-2, COVID-19, Delta, Omicron, severity markers

## Abstract

**Introduction:**

COVID-19 is caused by the severe acute respiratory syndrome coronavirus 2 (SARS-CoV-2), a virus notable for its rapid mutation rate, which has led to the emergence of various variants such as Delta and Omicron, each with potentially different levels of transmissibility and virulence. Therefore, this study aims to compare clinical charactheristics and markers associated with the severity of COVID-19 in hospitalized patients from western Mexico who were infected with the Delta and Omicron variants of SARS-CoV-2.

**Methods:**

This cross-sectional study involved 66 patients hospitalized for COVID-19, diagnosed by RT-qPCR. SARS-CoV-2 variants were identified through whole genome sequencing using the COVIDseq platform from Illumina. Upon admission, patients underwent a clinical history assessment, blood gas analysis, and blood biometry. Additionally, several tests and markers were measured, including the percentage of neutralizing antibodies, erythrocyte sedimentation rate (ESR), interleukin-6 (IL-6), tumor necrosis factor-alpha (TNFα), D-dimer, lactate dehydrogenase (LDH), C-reactive protein (CRP), and ferritin.

**Results and discussion:**

Patients hospitalized with the Omicron were found to be older, compared to those infected with the Delta (64 vs. 54 years, *p* = 0.006). Additionally, a higher proportion of male patients were observed in the Omicron compared to the Delta (*p* = 0.029). Both Omicron and Delta variants were associated with lymphopenia, although the lymphocyte count was lower in Omicron (0.9 vs. 0.56 10x^3^/L; *p* = 0.007). The COVID-GRAM scale indicated a high risk for severe disease in both groups, but the score was higher in Omicron compared to Delta (157 vs. 128 points; *p* = 0.0004). Patients infected with Omicron exhibited a lower percentage of neutralizing antibodies than those with Delta (35.99 vs. 81%; *p* < 0.05), regardless of their vaccination status. Among the markers assessed, globular ESR was found to be lower in Omicron compared to Delta (30.5 vs. 41.5 mm/h; *p* = 0.001), while ferritin levels were higher in patients infected with the Omicron (1,359 vs. 960.6 μg/L; *p* = 0.007). In patients with severe COVID-19, markers such as lymphopenia, neutralizing antibody levels, ferritin, and COVID-GRAM scores are elevated in the Omicron variant, while only the leukocyte count and ESR for the Delta variant.

## 1 Introduction

COVID-19, caused by the infectious agent SARS-CoV-2, manifests symptoms similar to severe flu, characterized by acute respiratory difficulty and acute inflammatory response. While most patients experience mild to moderate symptoms, 5–10% develop severe states, sometimes leading to death (1–3). The primary pathology presented in severe cases is acute respiratory impairment syndrome (ARDS), precipitated by a cytokine storm and an unregulated increase in the release of proinflammatory cytokines and chemokines, driving acute inflammation (4–6). Thus, elevated concentrations of biomarkers such as CRP, ferritin, ESR, D-dimer, fibrinogen, and LDH have been associated with increased critical risk and lethality in COVID-19 (7–9). The clinical characteristics and biomarkers reported have played a crucial role in the understanding and management of COVID-19, as they allow for early diagnosis, risk stratification, assessment of disease severity, monitoring response to treatments, and identifying the presence of complications, thus enabling personalized clinical decisions. Furthermore, the COVID-GRAM score is a tool that predicts the clinical risk of critical illness. Validated in the Chinese population, it showed predictive capacity for critical illness in Spain. When comparing the performance of different prognostic scales, it was the most accurate for identifying mortality in patients admitted with pneumonia (10–12).

SARS-CoV-2, a pleomorphic virus, possesses a single-stranded positive-stranded RNA genome ranging in size from 60 to 140 nm and weighing 8.4–12 kDa (3, 13). Due to continuous replication, RNA viruses like SARS-CoV-2 exhibit a high mutation rate in their genetic material, with RNA polymerase demonstrating a high corrective capacity. This results in a monthly mutation rate of 1–2 single nucleotide mutations for each viral lineage. However, these mutations may be non-synonymous, affecting the amino acid sequence of viral proteins (14, 15). Identifying multiple SARS-CoV-2 variants worldwide is a significant concern, as mutations have conferred increased transmissibility or virulence. Of particular interest are mutations in the S gene, which impact the S protein receptor binding domain (RBD). The S protein, binding to the ACE2 (angiotensin-converting enzyme 2) receptor, is crucial for viral entry into host cells. In addition, mutations in the S gene can also affect the effectiveness of neutralizing antibodies acquired through natural infection or vaccination. One notable example is the D614G mutation, present in many variants, which replaces aspartic acid with glycine at amino acid position 614. This mutation increases the infectivity of SARS-CoV-2, highlighting the significance of genetic variations in the virus' behavior and the challenges they pose for containment and control efforts (14, 16).

Due to the global emergence of various SARS-CoV-2 variants, the World Health Organization (WHO) has classified genetic variants into two categories according to their level of concern: Variants of Interest (VOI) and Variants of Concern (VOC). VOCs are of primary significant interest because they affect the level of transmissibility and virulence. Throughout the pandemic, five variants have been classified as VOC: Alpha, Beta, Gamma, Delta, and Omicron (14, 16–18). In Mexico, two VOCs, Delta and Omicron, and their respective sublineages, have predominated (19). The Delta variant, belonging to the B.1.617.2 lineage, was first detected in India in December 2020. Characterized by seven mutations in the S gene, the Delta variant exhibits increased transmissibility, higher viral load, and reduced antibody neutralization. The Omicron variant, part of the B.1.1.529 lineage, was first identified in South Africa in November 2021; its sublineages carry over 30 mutations in the S gene, contributing to its higher transmissibility (R0 = 10) and greater ability to evade neutralizing antibodies from vaccination or previous infection (14, 15, 17, 20, 21).

The serious consequences of COVID-19 result from two main pathological aspects: the direct effects of SARS-CoV-2 and the host's immuno-inflammatory response. Throughout the pandemic, multiple variants of SARS-CoV-2 have emerged worldwide. Thus, it is important to follow up epidemiologically and update which clinical parameters are affected by different SARS-CoV-2 variants and how COVID-19 evolves. Therefore, this study aims to identify which clinical characteristics and markers are associated with the Delta and Omicron variants of SARS-CoV-2 in hospitalized COVID-19 patients.

## 2 Materials and methods

### 2.1 Study design and participants

A cross-sectional study was conducted at the *Hospital Civil de Guadalajara “Dr. Juan I. Menchaca,”* Mexico.

**Figure 1:**
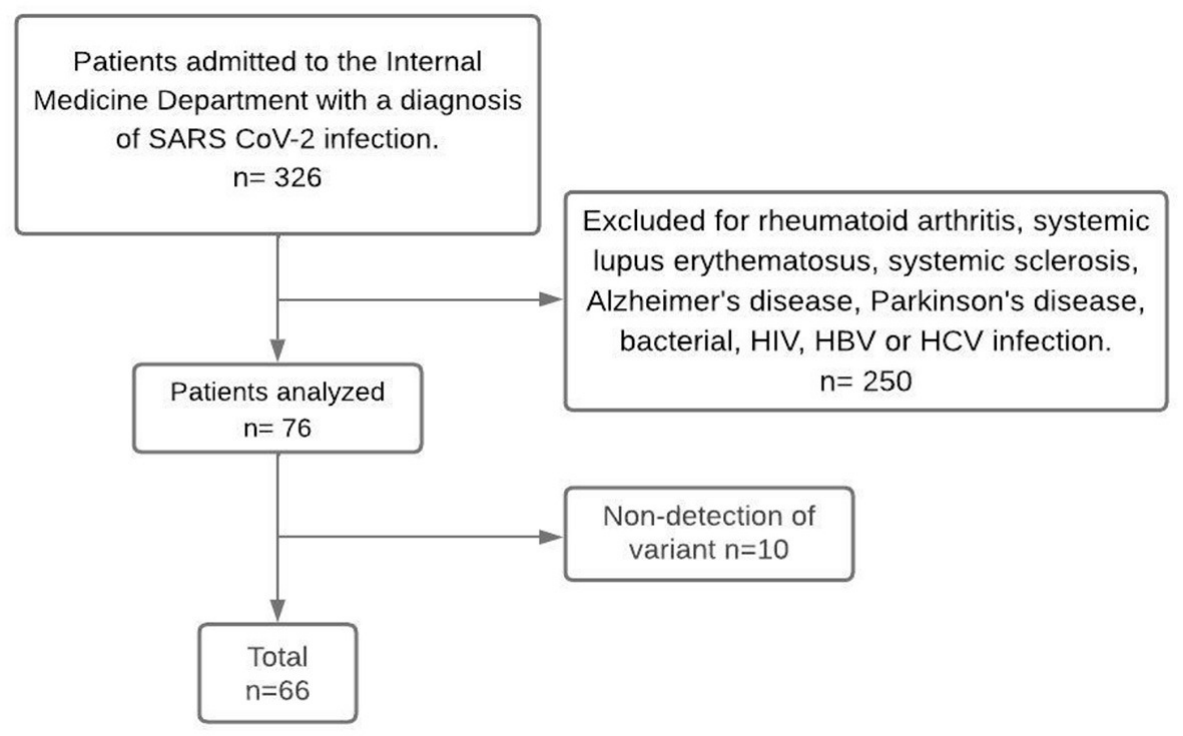
Flow chart of the participants. Non-detection of variant refers to samples with undetectable viral load that did not allow identification of the SARS-CoV-2 variant.

We included 66 patients from western Mexico (Colima, Jalisco, Michoacan and Nayarit) according to the inclusion criteria: >18 years old, both sexes, positive q-PCR for SARS-CoV-2 and hospitalized patients admitted to the Department of Internal Medicine between October 2021 and February 2022, which were classified as severe COVID-19 according to the World Health Organization criteria: SaO_2_ < 90%, respiratory rate > 30 and signs of severe respiratory distress (22); a single blood and nasopharyngeal sample was taken from each patient during hospital admission. All they confirmed to have COVID-19 by reverse transcriptase polymerase chain reaction (RT-PCR) through Ct detection (Cycle threshold) using the COVIFLU multiplex kit (GENES2LIFE, Mexico). Patients with any disease affecting marker concentrations and with a Ct > 29 in the RT-PCR were excluded, as the quality of the sequences did not allow for the identification of the SARS-CoV-2 variant (Figure 1). Sociodemographic characteristics such as age, sex, days to first symptom, comorbidities, type of comorbidities, vaccination status, dose, and previous COVID-19 disease were retrieved from the patient's clinical history. Additionally, the COVID-GRAM scale was used to determine the critical risk of each patient, taking into account the ten parameters: chest radiography abnormality, age, hemoptysis, dyspnea, unconsciousness, number of comorbidities, cancer history, neutrophil-to-lymphocyte ratio, lactate dehydrogenase, and direct bilirubin (12).

### 2.2 SARS-CoV-2 variant identification

Whole genome sequencing was used to identify SARS-CoV-2 variants. Viral RNA was extracted from nasopharyngeal swabs in a viral transport medium using the Quick RNA Viral Kits (ZYMO RESEARCH, USA). Sequencing was performed using the COVIDSeq test (Illumina, USA), following the manufacturer's instructions, which utilizes multiplexed amplification across the entire SARS-CoV-2 genome. Libraries were sequenced employing a MiSeq kit (Illumina, USA). The sequences were then assembled into complete genomes by the Illumina Basespace platform with the DRAGEN COVID Lineage Workflow program.

### 2.3 Laboratory test measurements

All patients in the study underwent blood gas analysis and blood biometry using the BC-5150 equipment (Mindray, China). The percentage of neutralizing antibodies directed against the S-protein RBD (receptor binding domain) of SARS-CoV-2 was performed using the cPass™ SARS-CoV-2 Neutralization Antibody Detection Kit (GenScript, USA). Markers associated with severity, such as CRP, D-dimer, and LDH, were measured using the AU5800 Serie Clinical Chemistry Analyzer (Beckman Coulter, USA), while ferritin levels were determined employing the BS-120 equipment (Mindary, USA). ESR was performed using the Wintrobe method. Additionally, TNF-α and IL-6 cytokines were analyzed using Bio-Plex Pro Human Cytokine 17-plex Assay (BIORAD, USA) and Human IL-6 High Sensitivity ELISA kit (Thermo Scientific^TM^, USA), respectively.

### 2.4 Statistical analysis

Statistical analysis was conducted using GraphPad Prism v5.0 and R software. Descriptive analysis included expressing nominal discontinuous variables (SARS-CoV-2 genetic variants) as frequencies, continuous variables with parametric distribution as means ± SD, and non-parametric variables as medians (minimum and maximum). The χ^2^-test was employed to compare proportions between study groups. The Student's *t*-test was used for parametric quantitative determinations, while the Mann-Whitney *U*-test was used for non-parametric determinations. Spearman's test was used for the correlation matrix. All statistical analyses were performed with *n* = 66, however outliers of the variables were identified using the Grubbs test. A *p*-value of < 0.05 was considered statistically significant.

### 2.5 Ethics

This study was approved by the ethics, biosafety, and research committee of the *Centro Universitario de Ciencias de la Salud* of the UDG (CI-03721) and by the research ethics committee of the *Hospital Civil de Guadalajara “Dr. Juan I. Menchaca”* (00095). It was conducted in accordance with the ethical standards described in the Declaration of Helsinki (1983).

## 3 Results

### 3.1 Epidemiological characteristics

A total of 66 patients with severe COVID-19 (Table 1) were included in the analysis, with a mean age of 58.38 years. Among these patients, 62.1% were men, and their hospital admission occurred, on average, 9 days after perceiving the first symptom. Notably, only 33.3% of the patients had received vaccination against SARS-CoV-2. In comparison, 65.2% had at least one comorbidity (such as systemic hypertension, diabetes, chronic renal disease, chronic heart failure, cerebral vascular disease, chronic obstructive pulmonary disease, asthma, and ex-smokers). Additionally, 51.51% of the patients had received corticosteroid treatment before hospitalization.

**Table 1:** Descriptive sociodemographic characteristics of the population.

**Variable**	**Total**	**Delta**	**Omicron**	***p***
	***n*** = **66**	***n*** = **39**	***n*** = **27**	
Age (years), X ± SD	58.3 ± 15.4	54.1 ± 15.3	64.5 ± 13.6	**0.006** ^**a**^
Grouped age (years), *n* (%)				
< 40	10 (15.2%)	9 (23.1%)	1 (3.7%)	0.084^b^
40–60	28 (42.4%)	16 (41%)	12 (44.4%)	
>60	28 (42.4%)	14 (35.9%)	14 (51.9%)	
Sex, *n* (%)				
Woman	25 (37.9%)	19 (48.7%)	6 (22.2%)	**0.029** ^**b**^
Man	41 (62.1%)	20 (51.3%)	21 (77.8%)	
Days at first symptom (days), X ± SD	9 ± 3.2	8.95 ± 3.1	9.07 ± 3.5	0.880^a^
Comorbidities, Yes (%)	43 (65.2%)	25 (64.1%)	18 (66.6%)	0.83^b^
SAH, Yes/No (%)	26 (39.4%)	12 (30.8%)	14 (51.8%)	0.085^b^
DM, Yes/No (%)	19 (28.78%)	11 (28.2%)	8 (29.6%)	0.90^b^
CKD, Yes/No (%)	1 (1.5%)	0 (0%)	1 (3.7%)	NA
CHF, Yes/No (%)	1 (1.5%)	0 (0%)	1 (3.7%)	NA
CVD, Yes/No (%)	2 (3%)	0 (0%)	2 (7.4%)	NA
COPD, Yes/No (%)	1 (1.5%)	1 (2.5%)	0 (0%)	NA
Asthma, Yes/No (%)	3 (4%)	2 (5.1%)	1 (3.7%)	0.78^b^
Arrhythmia, Yes/No (%)	1 (1.5%)	1 (2.5%)	0 (0%)	NA
Cancer, Yes/No (%)	1 (1.5%)	0 (0%)	1 (3.7%)	NA
Ex-smokers, Yes/No (%)	13 (19.7%)	7 (17.94%)	6 (22.2%)	0.66^b^
Smokers, Yes/No (%)	2 (3%)	2 (5.1%)	0 (0%)	NA
Corticosteroid prior to hospitalization, *n* (%)	34 (51.5%)	14 (21.2%)	20 (30.3%)	**0.002** ^**b**^
Vaccinated (%)	22 (33.3%)	10 (25.6%)	12 (44.4%)	0.111^b^
Two doses, Yes/No (%)	19 (86.4%)	7/10 (41.1%)	12/12 (50%)	0.078^c^

Med, Median; X, Medium; SD, Standard deviation; Max, Maximum; Min, Minimum; DM, Diabetes Mellitus; SAH, Systemic Arterial Hypertension; CKD, Chronic Kidney Disease; CHF, Congestive Heart Failure; CVD, Cerebrovascular Disease; COPD, Chronic Obstructive Pulmonary Disease.

^a^Student's T.

^b^Chi-squared.

^c^Fisher's Exact.

NA, does not apply statistical test; p-values are determined between Delta and Omicron; p-value bold: significant differences.

In the identification of SARS-CoV-2 variants among hospitalized patients, 39 (59.09%) of the patients were infected by the Delta variant, while 27 (40.91%) were Omicron. Significant differences (*p* = 0.006) were observed in the average age of patients admitted to the hospital for COVID-19: Omicron-infected patients had a mean age of 64.56 years, while those infected with the Delta variant had a mean age of 54.10 years. Furthermore, a higher proportion of Omicron infections were in males compared to Delta infections (*p* = 0.029). In addition, the administration of corticosteroids before hospitalization was higher in patients with omicron (*p* = 0.002).

A significant difference (*p* = 0.044) was observed in the total leukocyte count, with patients infected with the Delta variant (11.57 10x3/L) having a higher count compared to those infected with the Omicron variant (9.61 10x3/L). Furthermore, a significant decrease (*p* = 0.007) was found in the total lymphocyte count among patients infected with Omicron (0.56 10x3/L) compared to Delta (0.9 10x3/L). Regarding platelet concentrations, a higher count was identified in the Delta variant (259 10x3/L) compared to Omicron (188.8 10x3/L), *p* = 0.003. In addition, the COVID-GRAM score was higher in patients with Omicron compared to those with Delta (157 points vs. 128 points, *p* = 0.004). However, both scores corresponded to a high critical risk of the disease (low risk < 6, 6–128 moderate risk, and high risk >128). Other variables were outside normal ranges, but did not show statistical differences, including a decrease in SaO_2_, PaO_2_, and PaCO_2_ values, as well as an increase in procalcitonin, total neutrophils and their percentage (refer to Table 2).

**Table 2:** Laboratorial characteristics of patients infected by Delta and Omicron variants.

**Variable**	**Total**	**Delta**	**Omicron**	***p***
	***n*** = **66**	***n*** = **39**	***n*** = **27**	
Ct, X ± DE	26.3 ± 4.1	25.6 ± 4.2	27.2 ± 3.8	0.138^a^
Viral load (CT), *n* (%)				
High (< 25)	23 (35%)	16 (41%)	7 (26%)	0.276^c^
Medium (25–30)	29 (44%)	17 (43.6%)	12 (44.4%)	
Low (>30)	14 (21.2%)	6 (15.4%)	8 (29.6%)	
Blood gases, Med (Min-Max) or X ± DE				
SaO_2_ (%)	61 (48–97)^§^	84.5 (48–97)^§^	86 (60–97)^§^	0.166^b^
pH	7.4 (7.1–7.6)	7.4 (7.2–7.6)	7.4 (7.1–7.5)^†^	0.501^b^
PaO_2_ (mmHg)	61 (21–249)^§^	64 (21–249)^§^	60 (24–92)^§^	0.661^b^
PaCO_2_ (mmHg)	33 (10–89)^§^	33 (10–52)^§^	33 (25–89)^§^	0.675^b^
hCO_3_ (mEq/L)	22.2 ± 5.4	22.1 ± 5.65	22.2 ± 5.0	0.989^a^
Oxygenation devices				
No oxygen	2 (3.1%)	1 (2.5%)	1 (3.7%)	
Nasal tips	8 (12.1%)	3 (7.6%)	5 (18.5%)	
Mask	12 (18.2%)	7 (17.9%)	5 (18.5%)	0.654^c^
High flow	25 (37.9%)	17 (43.6%)	8 (29.6%)	
VMI	19 (28.7%)	11 (28.2%)	8 (29.6%)	
Blood biometrics, Med (Min-Max) or X ± DE				
Total leukocytes (10x^3^/L)	10.7 ± 4.1^†^	11.5 ± 4.6^†^	9.6 ± 3.0	**0.044** ^**a**^
Total lymphocytes (10x^3^/L)	0.7 (0.2–2.4)^§^	0.9 (0.3–2.2)^§^	0.5 (0.2–2.4)^§^	**0.007** ^**b**^
Percentage of lymphocytes (%)	7.6 (1.73–35.0)^§^	8.9 (2.2–35.0)^§^	5.9 (1.7–26.2)^§^	0.088^b^
Total neutrophils (10x^3^/L)	9.3 ± 3.9^†^	10.0 ± 4.3^†^	8.3 ± 3.1^†^	0.083^a^
Percentage of neutrophils (%)	86.8 (28.7–96.2)^†^	85.6 (28.7–95.8)^†^	87.5 (61.3–96.2)^†^	0.285^b^
Platelets (10x^3^/L)	224.0 (46.7–487.2)	259 (102.3–487.2)	188.8 (46.7–407)	0**.003**^**b**^
Procalcitonin (ng/mL)	0.2 (0.07–53)^†^	0.21 (0.07–1.1)^†^	0.29 (0.07–53.0)^†^	0.363^b^
GRAM, Med (Min–Max)	140 ± 31^†^	128 ± 24^†^	157 ± 33^†^	**0.0004** ^**a**^

Med, Median; X, Medium; SD, Standard deviation; Max, Maximum; Min, Minimum.

^a^Student's T.

^b^Mann Whitney U.

^c^Chi-squared; Normal values (VN), SaO_2_ (%): 90–95, pH: 7.35–7.42, PaO_2_ (mmHg): 70–100, PaCO_2_ (mmHg): 35–45, hCO_3_ (mEq/L): 19–25; LeuT (10x^3^/L): 4.4 −11.3, LinT (10x^3^/L): 1–4.8, Lin (%): 18 −45, NeuT (10x^3^/L): 1.8 −7.7, Neu (%): 40 −85, Plaq (10x^3^/L): 150 −450, Proc: < 0.1, GRAM: 6–128 moderate risk.

^§^Below VN.

^†^Above VN; viral load groups were selected according to the 33rd and 66th percentile; p-values are determined between Delta and Omicron; p-value bold: significant differences.

### 3.2 Percentage of neutralizing antibodies

**Figure 2:**
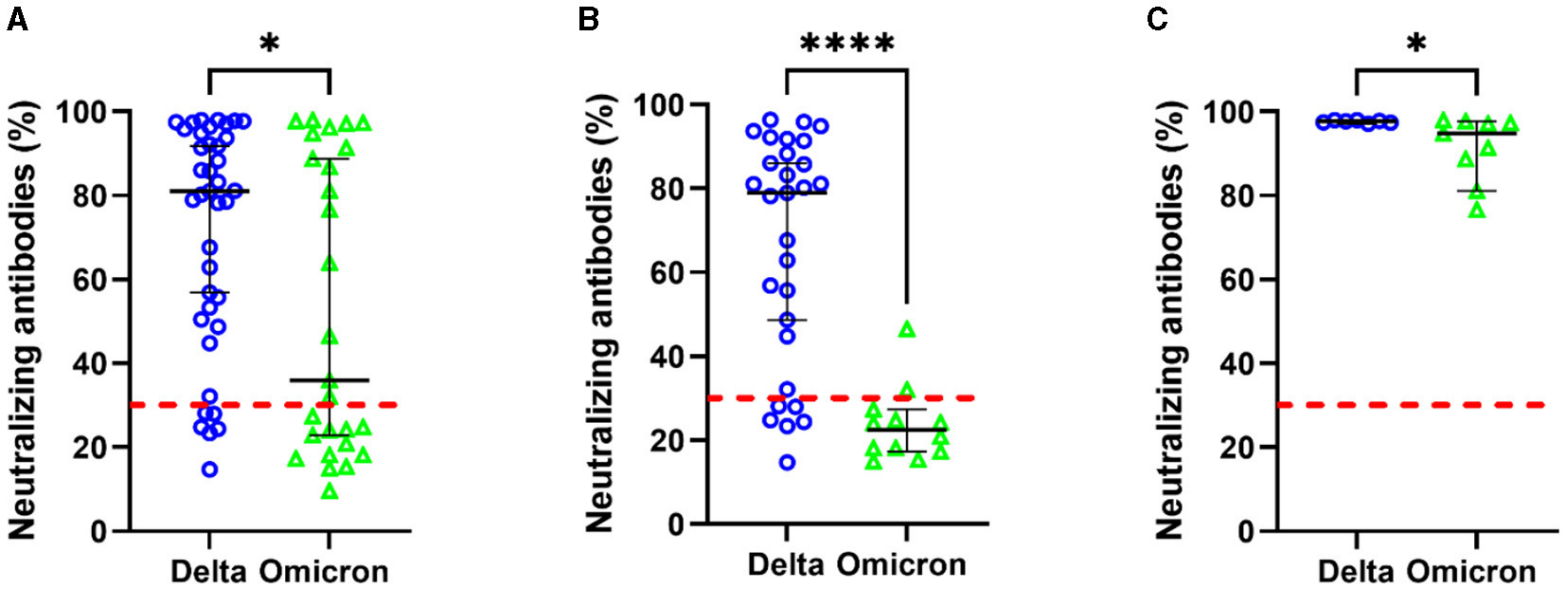
Percentage of neutralization antibodies according to SARS-CoV-2 variants. Patients' total **(A)**, unvaccinated patients **(B)**, and vaccinated patients **(C)**. Statistical comparisons between groups were determined using the Mann–Whitney *U*-test. *p < 0.05; ****p < 0.0001; a value >30 indicates the limit of the presence of antibody titers, shown by the red line.

Significant differences were found in the percentage of neutralizing antibodies between both study groups. Patients infected with the Delta variant had higher levels of neutralizing antibodies than those infected with Omicron (81 vs. 35.9%, *p* = 0.03; refer to Figure 2A). Given the high percentage of unvaccinated patients and the fact that they reported no previous COVID-19 illness, the percentage of neutralizing antibodies was analyzed in both vaccinated and unvaccinated groups. In unvaccinated patients, those infected with the Delta variant had higher levels of neutralizing antibodies compared to those infected with Omicron (78.95 vs. 24.09%, *p* = 0.00016; refer to Figure 2B). In vaccinated patients, levels were higher in Delta (97.73%) compared to Omicron (94.80%), with a value of *p*-value of 0.031 (refer to Figure 2C). However, it was not possible to perform the analyses according to the time and type of vaccine, as the patients were hospitalized as emergency patients and did not have the exact information.

### 3.3 Severe COVID-19 markers

**Figure 3:**
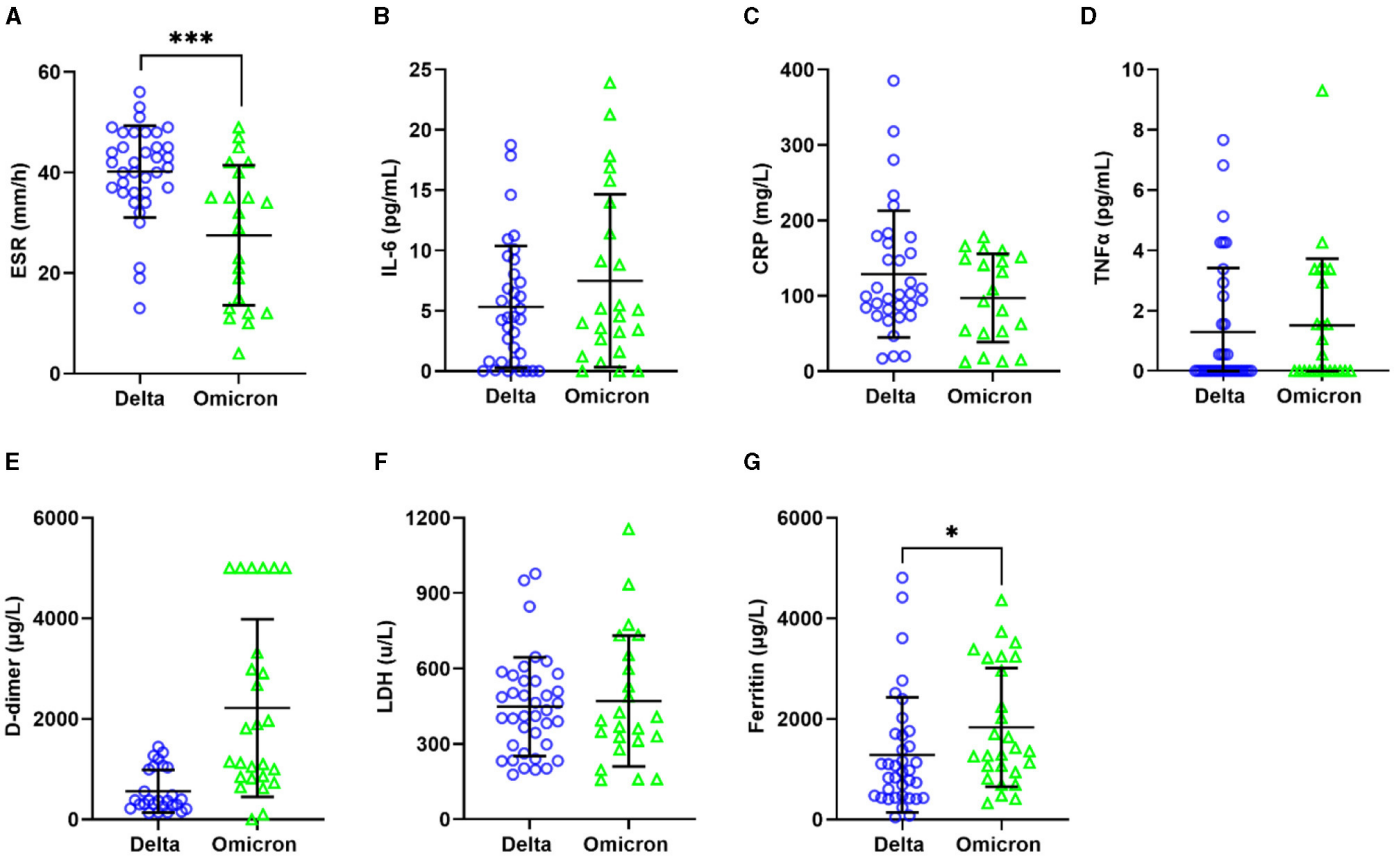
Relationship between severity biomarkers and SARS-CoV-2 variants. ESR **(A)**, IL-6 **(B)**, CRP **(C)**, TNFα **(D)**, D-dimer **(E)**, LDH **(F)**, and Ferritin **(G)**. Statistical comparisons between groups were determined using the Mann–Whitney *U*-test. *p < 0.05; ***p < 0.001.

Regarding severity markers, significant differences were found only in ESR and ferritin levels. Patients infected with the Delta variant presented a higher median ESR (41.5 mm/h) compared to those with the Omicron variant (30.5 mm/h), *p* = 0.0015 (Figure 3A). Additionally, the median ferritin level was higher in patients with the Omicron variant (1,359 μg/L) with respect to those with the Delta variant (960.6 μg/L), *p* = 0.035. No significant differences were observed for LDH, CRP, IL-6, TNFα, and D-dimer. However, the Delta variant showed a slight increase in median CRP (128.9 mg/L), and a decrease in D-dimer (371 μg/L), LDH (447.9 u/L), TNFα (0 u/L), and IL-6 (4.45 pg/mL), compared to Omicron variant (LDH: 470.6 u/L, CRP: 97.21 mg/L, D-dimer: 1,480 μg/L, TNFα: 0.27 u/L and IL-6: 4.81 pg/mL; Figures 3B–G). A multiple linear regression model adjusted for age, sex, comorbidities, and SARS-CoV-2 variants was performed to observe their influence on the significant results. The analysis showed that only the ferritin value obtained was significantly related to the sex variable (β = −1,950.59; SE = 727.32), while the other variables were related to the SARS-CoV-2 variants (Table 3).

**Table 3:** Multiple linear regression models adjusted for age, sex, comorbidities and SARS-CoV-2 variants.

**Adjusted model**	**Total leukocytes (β; SE)**	**Total lymphocytes (β; SE)**	**COVID-GRAM (β; SE)**	**Neutralizing antibodies (β; SE)**	**ESR (β; SE)**	**Ferritin (β; SE)**
Intercept	10.85 (2.22)^***^	1.05 (0.28)^***^	100.43 (14.97)^***^	54.81 (16.44)^***^	32.58 (7.20)^***^	4,134.54 (1,466.17)^**^
Age	0.0006 (0.03)	−0.003 (0.004)	0.33 (0.24)	0.16 (0.26)	0.05 (0.11)	−16.88 (23.3)
Sex (Woman)	−0.297 (1.1)	0.27 (0.13)	3.59 (7.39)	2.29 (8.15)	3.74 (3.77)	−1,950.59 (727.32)^**^
Comorbidities (Yes)	1.27 (1.08)	0.05 (0.13)	12.77 (7.22)	9.53 (7.99)	0.78 (3.69)	−106.71 (713.20)
Variant (Omicron)	−2.06 (1.12)	−0.16 (0.14)	24.96 (7.65)^**^	−19.94 (8.31)^*^	−9.60 (3.88064)^*^	−708.46 (741.62)

β, beta regression coefficient; SE, Standard error.

^*^p < 0.05.

^**^p < 0.01.

^***^p < 0.001.

**Figure 4:**
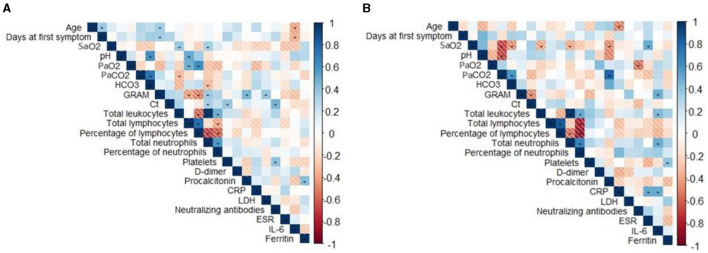
Correlation matrix of demographic and laboratory data. Correlation matrix for Delta **(A)** and Omicron **(B)**. Blue and red color indicate positive and negative correlation, respectively. Statistical comparisons between groups were determined using the Spearman test. *p < 0.05. Ct, Cycle threshold; LDH, lactate dehydrogenase; CRP, C-reactive protein; ESR, erythrocyte sedimentation rate.

Finally, correlations between clinical variables were analyzed for patients infected with the Delta and Omicron variants (Figure 4). In the Delta variant group, age showed a positive correlation with the number of days to the onset of the first symptom (*r* = 0.36, *p* = 0.02) and COVID-GRAM score (*r* = 0.24, *p* = 0.03), and negative associations with IL-6 levels (*r* = −0.41, *p* = 0.02). Furthermore, oxygen saturation correlated positively with total leukocyte count (*r* = 0.37, *p* = 0.02) and neutrophil count (*r* = 0.33, *p* = 0.04). Significant positive correlations were also observed between Ct values and the percentage of neutralizing antibodies (*r* = 0.46, *p* = 0.003), platelets (*r* = 0.38, *p* = 0.01), and total neutrophil count (*r* = 0.32, *p* = 0.05). In the Omicron variant group, age was negatively correlated with CRP levels (*r* = −0.51, *p* = 0.02). Oxygen saturation showed negative correlations with total leukocytes (*r* = −0.40, *p* = 0.04), total neutrophils (*r* = −0.40, *p* = 0.03), and procalcitonin (*r* = −0.49, *p* = 0.04), and positive correlations with ESR (r = 0.50, p = 0.01). Furthermore, IL-6 correlated positively with total leukocytes (*r* = 0.43, *p* = 0.03), total neutrophils (*r* = 0.49, *p* = 0.01), CRP (*r* = 0.50, *p* = 0.05), and COVID-GRAM score (*r* = 0.48, *p* = 0.01).

## 4 Discussion

This study compares clinical characteristics and markers associated with severity in patients hospitalized with COVID-19 for the Delta and Omicron variants of SARS-CoV-2. Our analysis revealed that different variables, including age, sex, lymphocyte count, COVID-GRAM score, percentage of neutralizing antibodies, ESR, ferritin, and IL-6, were significantly associated with a specific variant of SARS-CoV-2 infection.

The average age of hospitalized patients with the Omicron variant was 64 years. In contrast, those infected with the Delta variant had a lower average age of 54. Additionally, men showed a higher infection rate across both variants. Advanced age has been associated with a higher prevalence of comorbidities due to the natural aging process and increased susceptibility to respiratory illnesses due to immunosenescence (23). This finding aligns with Modes et al., who reported a similar trend, with patients infected with the Omicron variant being older (66 years) compared to those with Delta (60 years) (24). Furthermore, age emerges as a risk factor for pneumonia during the Omicron wave, with an odds ratio (OR) of 1.046 (25). Notably, *in-vitro* studies have shown that alveolar type 2 cells from older patients express higher ACE2 receptors, which facilitates viral entry, compared to cells from children, although TMPRSS2 expression remains unchanged (26).

A meta-analysis conducted by Fabiao et al. revealed that being male was associated with a relative risk of 1.29 compared to females, indicating a higher risk of COVID-19 mortality in males regardless of age (27). This sex-based difference in susceptibility to severe outcomes could be attributed to X-chromosome gene expression and estrogen production variations, which may influence the immune response, particularly in women (28). Baristaite and Gurwitz further demonstrated that the female sex hormone estradiol can reduce the concentration of ACE2 and TMPRSS2 mRNA in human lung epithelial A549 cells (29).

In blood biometry, both leukocyte and lymphocyte counts were increased in patients infected with the Delta variant; however, lymphopenia was observed in hospitalized patients with both variants. Kilic et al. found that lymphopenia poses an 8.9-fold risk for severe COVID-19 and a 12.4-fold risk for mortality (30). Additionally, Diao et al. identified a negative correlation between elevated concentrations of proinflammatory cytokines (IL-6, TNF-α, and IL-10) and lymphocyte concentration. They also observed increased expression of programmed cell death protein 1 (PD-1) and mucin domain 3 (Tim-3) in CD4 and CD8 T cells (31).

A notable difference was observed in platelet concentration, with higher counts in patients infected with the Delta variant compared to those with the Omicron variant. However, a study conducted in Japan reported an increase in platelet levels among patients infected with the Omicron variant (32). Platelets, small anucleated cell fragments derived from megakaryocytes, play a pivotal role in initiating the initial cellular response to vascular injury (33). Furthermore, the Delta variant has been associated with a slightly increased prevalence of pulmonary embolism compared to patients infected with the Omicron variant (34).

The COVID-GRAM score was notably higher in patients infected with the Omicron variant, indicating an elevated risk of COVID-19 disease severity. The COVID-GRAM score is derived from 10 variables that collectively predict the clinical risk associated with COVID-19 (12).

The Omicron variant presented a lower percentage of neutralizing antibodies compared to the Delta variant, particularly in neutralizing the receptor-binding domain (RBD) of SARS-CoV-2 wild type (WT). This disparity was observed across both vaccinated and non-vaccinated patients. Neutralizing antibodies are crucial proteins produced by the humoral immune system, which bind to epitopes of the pathogen to inhibit or neutralize its infectivity (35). Studies have indicated that the Omicron variant displays enhanced evasion of neutralizing antibodies from both convalescent patients and individuals vaccinated with Pfizer and AstraZeneca platforms (36). This evasion has been linked to mutations in S protein, including those identified in bioinformatics analyses (S304, S371L, S373P, and S375F), which are believed to enhance the variant's interaction with the ACE2 receptor, consequently increasing its transmissibility (37). Although the patients mentioned during the clinical interview that they had not been previously infected by COVID-19, neutralizing antibodies were found in the unvaccinated patients. A study in Mexico reported that 49.6% of young adults had no previous SARS-CoV-2 infection but were seropositive for anti-SARS-CoV-2, and 48.1% had neutralizing antibodies (38).

The erythrocyte sedimentation rate (ESR) displayed a higher mean in patients hospitalized for the Delta variant. ESR is a diagnostic test that measures the rate at which erythrocytes sediment due to the presence of proteins with inflammatory activity (39). While direct comparisons of this parameter between the Delta and Omicron variants are limited, Li et al. reported that patients infected with the Omicron variant have an ESR within normal ranges (40).

Ferritin levels were higher in patients infected with the Omicron variant. Ferritin is an acute-phase inflammatory protein that serves as an indicator of cellular damage (41). Alroomi et al. established that ferritin is a predictive marker for COVID-19 severity and mortality, with concentrations exceeding 1,000 μg/L correlating with an 8.48-fold higher likelihood of pneumonia (42).

The IL-6 cytokine serves as a marker for COVID-19 severity, showing a negative correlation with age in patients infected with the Delta variant and a positive correlation with CRP, COVID-GRAM score, total leukocyte count, and neutrophils in those infected with the Omicron variant. Coomes and Haghbayan conducted a meta-analysis revealing that IL-6 levels are 9.6 times higher in severe COVID-19 cases and are associated with adverse outcomes (43). Bioinformatics studies have indicated that Delta variant proteins have higher epitopes that promote IL-6 expression (44). Moreover, the Delta variant has been reported to induce higher concentrations of NF-kB, a key transcription factor for pro-inflammatory cytokine expression (45). Additionally, macaques infected with Delta variant pseudoviruses showed higher IL-6 expression than those infected with BA.1 and BA.2 Omicron variants (46).

The main strengths of the study included the identification of SARS-CoV-2 variants by sequencing, the focus on patients hospitalized for COVID-19, and the determination of key severity markers. Additionally, the results were not influenced by demographic characteristics such as age, sex and comorbidities. However, the study had several limitations: pre-hospitalization corticosteroid treatment in some patients, prolonged time to hospitalization after the symptom onset, lack of information on the type and timing of vaccination, limited sample size, absence of a healthy control group, and insufficient data on prior COVID-19 infections and the corresponding SARS-CoV-2 variants.

## 5 Conclusion

In conclusion, our results suggest that most severity markers are higher in the Omicron variant of SARS-CoV-2 compared to Delta in patients hospitalized for severe COVID-19. Risk factors associated with COVID-19 severity, such as older age and male gender, were more prevalent in Omicron-infected patients; however, these do not influence severity markers. Additionally, Omicron-infected patients exhibited higher lymphopenia, lower neutralizing antibody levels, elevated ferritin values, and higher COVID-GRAM scores, indicating a potentially less effective immune response against SARS-CoV-2 and a more intense clinical presentation. Despite a higher vaccination rate among patients infected with the Omicron variant, severe cases of COVID-19 were still observed. For the Delta variant, only the total leukocyte count and ESR were elevated. Further studies are needed to confirm these results, as continued monitoring of emerging variants and a thorough understanding of their clinical and biological implications remain crucial.

## Data Availability

The original contributions presented in the study are included in the article/supplementary material, further inquiries can be directed to the corresponding author.
